# Shifting the paradigm in outreach to under-represented groups

**DOI:** 10.15694/mep.2018.0000286.1

**Published:** 2018-12-17

**Authors:** Ike Okafor, Lauren Phillips

**Affiliations:** 1 University of Toronto Faculty of Medicine; 2 Department of Laboratory Medicine and Pathobiology

**Keywords:** diversity, inclusion, medicine, research, equity, equality, empowerment, support, underrepresentation, mentorship, opportunity, collaborative, longitudinal, leadership, medical school, clinical, physicians, representation, AAMC, MCAT

## Abstract

This article was migrated. The article was marked as recommended.

The Community of Support (COS) is a longitudinal and collaborative initiative that enables students who are Indigenous, Black, Filipino, economically disadvantaged, or who self-identify with having a disability to join and receive support at any stage of their medical school journey. The goal of COS is to increase diversity in the fields of research and medicine, as a diverse physician taskforce is essential to meeting the needs of Canada’s patient population. Our program supports students at various points in their academic careers, beginning from first year of undergrad to end of PhD and into the workforce. We offer a variety of support systems that aim to address gaps and empower students. Our three-pronged approach provides COS members with support at the levels of i) admissions information, ii) mentorship and experiential opportunities, and iii) application support (including MCAT prep). Over the past 3 years, we have grown to include over 1,100 participants at various stages of their medical school journeys, from first year undergraduate students to university graduates from institution across Canada. As a result, in just three years, we have supported over 80 students with successful admissions to medical school, and alumni from CoS are now represented in 11/15 Canada’s medical schools, with a growing number of US schools, such as Yale University, George Washington, Michigan State and Wayne State.

## Introduction

The lack of representation and diversity in the medical field is a multifaceted issue that impacts communities nationwide. A diverse physician task force is essential to meeting the needs of Canada’s dynamic and multicultural population ( Cohen, Gabriel and Terrell, 2002; Figueroa, 2014). The disparity between the needs of Canada’s population and current physician workforce can be attributed to socioeconomic barriers faced by minority populations attempting to navigate the premed journey in addition to systemic issues in the education system ( James, 2012; Young *et al.*, 2017). One potential avenue to addressing this issue is to provide support that helps to alleviate societal and economic disadvantages faced by applicants at all points in their medical school journey. The Community of Support (CoS) is a longitudinal and collaborative initiative that supports students who are Indigenous, Black, Filipino, and economically disadvantaged or who self-identify with having a disability at every stage of their medical school journey. This responds to a call for a broader conceptualization of diversity at Canadian medical schools ( Young *et al.*, 2012). CoS works towards identifying and eliminating a variety of socioeconomic and cultural barriers, increasing diversity in all aspects.

Previous systematic reviews discuss a limited application pool as the rate limiting step to increasing diversity in medicine ( James *et al.*, 2012; Simone *et al.*, 2018). CoS addresses this hindrance with a unique approach that supports students at every stage in their medical school journey, which in turn prevents a “leaky pipeline” which refers to the loss of well qualified applicants due to lack of support and resources available to them at various stages in their medical school journey( James *et al.*, 2012). By providing students with a space to i) self-identify, ii) join at any stage, and iii) supporting them longitudinally, we can better support the limited pool of applicants. Socioeconomic barriers faced by minority communities can make components of the application process i.e.; writing the MCAT, and obtaining unpaid research/ volunteer opportunities inaccessible. We aim to alleviate these barriers by providing students with support at the levels of admissions, mentorship, and applications. CoS embodies the best practices highlighted by the American Association of Medical Colleges (AAMC), which had identified building a support network, cultivate personal attributes, provide access to information, and facilitate enrolment in premedical programs ( AAMC, 2015) as key success factors in enhancing access for Black Males in medicine.

Over the past 3 years, we have grown to include over 1,100 participants at various stages of their medical school journeys, from first year undergraduate students to university graduates from institution across Canada. As a result, in just three years, we have supported over 80 students with successful admissions to medical school, and alumni from CoS are now represented in 11/15 Canada’s medical schools, with a growing number of US schools, such as Yale University, George Washington, Michigan State and Wayne State.

## Objectives

The CoS aims to achieve the following;

• Foster a community of equity, inclusion, and diversity through excellence
• Reduce financial barriers by providing students with free resources (MCAT preparation courses, application support, mentorship)
• Provide students with highly valuable paid and volunteer opportunities in the fields of medicine and research
• Enable students to gain valuable skills to further develop their potential as future health professionals
• Enable students to see themselves reflected amongst medical students and physicians from similar backgrounds
• Facilitate environments of peer mentorship and leadership within marginalized communities

## Methods

The Community of Support (COS) serves as an umbrella program for numerous initiatives at the levels of academics, research, extracurriculars, and admissions that all work towards garnering a diverse physician task force and reducing the barriers associated with the medical school application process.

Student Application Support Initiative (SASI) + mock interviews

A mentorship program through which premedical students are paired with medical students to guide them through the medical school application process. This initiative is open to COS premedical students across the country. Medical student mentors provide mentees with advice in regard to course selection, extracurricular activities, and opportunities to best support their unique application package. Mentors provide mentees with feedback on their med school applications. As applicants receive interviews, they are connected to mentors from specific schools to help them prepared based on the specific school format. Canadian medical schools such as Dalhousie, McMaster, and McGill. These institutions also provide mentor support for CoS students with interview preparations taking place at each respective university making the SASI initiative accessible for all students regardless of geographical location.

### MCAT Student Support Program (MSSP)

A free 11-week program held from May to July to facilitate MCAT preparation. Students are provided with medical school tutors for 6 hours per week reviewing all MCAT ^2015 *^subjects: Biological Sciences, Biochemistry, Critical Analysis and Reasoning Skills, Inorganic and Organic Chemistry, Physics, and Psychology and Sociology. Full length practice tests from AAMC, Next Step Test Prep, and Khan Academy are utilized to gage student progress.

### Biostatistics Enrichment Program (BioStats)

A summer intensive program designed to connect premedical students to Rhode scholars and provide them with the bioinformatics skill to analyze large population studies. This is an excellent opportunity for students to obtain research publications and garner bioinformatics knowledge that can further develop their potential as future health professionals.

### Research Application Support Initiative (RASI)

A year long mentorship program through which premedical students with a passion for research are paired with graduate and/or medical students. Mentors provide mentees with application advice/ revision for graduate school programs and research opportunities. Furthermore, the CoS has affiliations with several research institutions to provide volunteer, and paid part time and full-time opportunities. To ensure longevity and success, students are provided with support and guidance after securing one of the aforementioned positions to maximize their research productivity.

### Additional opportunities

#### Doc Talks

Weekly sessions are held during summer where physicians share their medical journey with students and students have the opportunity to network and obtain advice.

#### Webinars

To accommodate students across the province and country informative seminars are held online. These seminars would cover topics such as school specific admission criteria and application support.

#### Conferences

The CoS hosts an annual IGNITE conference during which students have the opportunity to network with physicians/ medical students, get information on admissions requirements for Canadian, US and Caribbean schools.

#### CoS Chapters

The CoS began as a University of Toronto based initiative that has expanded to several universities across the country. Student-led CoS initiatives are currently active in 11 universities across the country; McMaster University - Black Aspiring Physicians of McMaster (BAP-Mac), Ottawa University - Association of Black Aspiring Physicians (ABAP), University of Ontario Institute of Technology- Black Physicians of Tomorrow (BPT), University of Toronto Scarborough - Future Black Physicians (FBP), University of Toronto St. George - Black Doctors of Tomorrow, UTM - Aspiring Physicians of Tomorrow, University of Toronto graduate students - Black Researcher’s Initiative to Empower (BRITE), Western - Western Future Black Physicians, York University - Future Black Physicians Initiative, Queen’s University - Association of Black Aspiring Physicians (ABAP), Wilfrid Laurier University Future Black Physicians.

Chapters provide peer mentorship, seeing yourself reflected with like-minded peers and leadership opportunities. Furthermore, numerous chapters have received provincial funding to run STEM mentorship programs for high school students.

## Discussion

CoS presents a unique and successful approach to addressing the lack of diversity in medical school. This lack of diversity has been attributed to; a limited application pool, financial burdens associated with the application process, and inaccessibility of traditional premedical opportunities.

A distinguishing aspect of CoS is our longitudinal and collaborative approach that allows prospective medical students to obtain support regardless of their stage in the medical school journey, and their school affiliation or status as a student. Diversity driven initiatives need to pivot away from traditional approaches of supporting the “talent 5%” where only small number of students are supported longitudinally. This approach is highly ineffective because there are larger constituencies of students from priority communities which go unsupported. Unrepresented students must have the option to obtain multifaceted support at any stage in their medical school journey. Furthermore, our collaborative nature makes this initiative high accessible to students across the country. To date, 11 CoS chapters have been established across the country and have been essential to the longevity and accessibility of the program. Certain medical schools across Canada have also begun collaborating on program development and supporting the current applicants. In order to impact systematic change, universities ***must*** collaborate. It is more practical and a social responsibility to look past the narrow confines of where students study. Collaboration is a core competency expected in physicians, therefore it is imperative that medical schools model this with their programming to support a diverse application pool. Additionally, collaborative programs aid in the reduction of overall costs and are financially stable. With multiple collaborating partners, you can more efficiently support a large prospective pool economically by leveraging resources - which reduces the overall cost of programming. Moreover, students supported by collaborative partners may become future medical student and/or residents in the future. A direct result of collaboration is the high impact that can be achieved in a short period of time. In the past three years, CoS has significantly increased the diversity in the UofT medical school student body (roughly 9% of entering class from 2018) and has impacted diversity at other Canadian and US schools.

Additionally, under a proportional universalism model- multiple groups (racial/ethnic, financial need, etc.) can be supported based on their specific needs. Supporting multiple groups differently is key because there are a wide variety of underrepresented groups that face barriers to becoming physicians. Programming should reflect general support required i.e application support, research opportunities, support etc of any underrepresented group but also be mindful of diverse needs). CoS exemplifies this approach by providing students general support as well as highly specific support depending on their demographic and financial needs e.g. MSSP, RASI, SASI etc. As a model for **shifting the paradigm in outreach to under-represented groups** we aspire to achieve and inspire other institutions to make systematic changes in their approach to diversity and inclusion initiatives.

## Take Home Messages

• A longitudinal and collaborative initiative that supports students at any point in their medical school journey is essential to increasing diversity
• Collaboration between universities is pivotal to the success of diversity initiatives and impacts in specific school application pools. Collaboration is cheaper and contributes to greater impact in a short period of time
• In collaborating, schools should focus on social responsibility of an overall increase in populations access to medical school generally, and not at their specific institution
• Support to reduce the socioeconomic barriers faced by applicants are key and requires more attention

## Notes On Contributors

Lauren Phillips is a graduate student in Department of Laboratory Medicine and Pathobiology, University of Toronto Faculty of Medicine. Lauren is also a member of COS and has a work study role where she is enhancing supports for COS students seeking research and mentorship opportunities.

Ike Okafor works in the Faculty of Medicine at the University of Toronto, where he develops and leads pre-med support initiatives and works with other departments to enhance accesbility to medical school.
