# Supplementary material for: Shifting the paradigm in outreach to under-represented groups
Source: MedEdPublish (2016). 2018 Dec 17;7:286. [Version 1] doi: 10.15694/mep.2018.0000286.1 (PMC10711985; doi:10.15694/mep.2018.0000286.1)
Supplement: Peer review history

# Reviewer report: Reviewer response for version 1

**DOI:** 10.21956/mep.19737.r29580

**Published:** 2019-01-05

**Authors:** P Ravi Shankar

**Affiliations:** 1 American International Medical University

This review has been migrated. The reviewer awarded 4 stars out of 5 I enjoyed reading this well-written article about the community of support (CoS) at Canadian medical schools.. The comprehensive nature of the program and the fact that it is available throughout a student’s course of study makes it more effective. I am interested in knowing more details about how students are selected for participation in CoS. Also there are CoS programs at different universities in Canada. Are these run as independent programs or is there a centralized administrative mechanism? Similar programs have been organized in other countries and locations and a description of some of these have been published in this journal. The article will be of broad interest to medical educators. There are a few spelling errors which could be corrected.

Reviewer Expertise:

NA

No decision status is available

# Reviewer report: Reviewer response for version 1

**DOI:** 10.21956/mep.19737.r29579

**Published:** 2019-01-02

**Authors:** Hassaan Waqar

**Affiliations:** 1 St Helens and Knowsley Teaching Hospitals NHS Trust – Lead Employer

This review has been migrated. The reviewer awarded 3 stars out of 5 Overall, this appears to be a well written article regarding an initiative to increase diversity within medical schools. The abstract concisely summarizes the article and accurately describes the information contained within. The introduction neatly encapsulates the problem of reduced diversity within medical schools and offers a solution which is subject of the paper. The methods section details the different strands of the Community of Support (CoS), however stops short of detailing how these strands were set up and any challenges that may have been faced. For example, the different chapters of the CoS are mentioned, however the hurdles faced in setting up these chapters are not explored. The discussion section notes a previous approach to increasing diversity and compares the CoS approach to this, noting how the weaknesses of the prior approach have been improved. However, the effectiveness of the different strands of the CoS approach are not explored and which if any of the strands were particularly effective in increasing diversity. The effectiveness of the CoS approach is mentioned with regards to one medical school in Canada. The impact on diversity is noted on other medial schools, however this is not explored or quantified. Additionally the discussion section details that collaboration amongst medical schools is a necessity in order to increase diversity, however the barriers that may exist to increasing collaboration are not addressed nor explored. The take home messages are short and concise, however there is little information as to how the take home messages may be implemented.

Reviewer Expertise:

NA

No decision status is available

# Reviewer report: Reviewer response for version 1

**DOI:** 10.21956/mep.19737.r29581

**Published:** 2018-12-25

**Authors:** Ken Masters

**Affiliations:** 1 Sultan Qaboos University

This review has been migrated. The reviewer awarded 2 stars out of 5 This is an interesting paper on broadening the intake into Canadian medical schools, and following through with their student career. I feel, however, that the paper is extremely broad, and greater value would be added if the authors could drill down into the detail. For example: • Demographics: The authors give the five demographic groups only, and indicate the number of students in total who have been given assistance, but it would be useful if we could see a deeper breakdown of the figures per group and also per year and the type of assistance that was given. This would allow the reader to have more insight for evaluating the success of the project.• Initiatives: While the list of initiatives is impressive, again, there is no detail on how many of which people benefited, and what the results were. These details really are necessary to gauge the scope and impact of the project.• Problems: No project is without problems, and a project of this type must have had huge obstacles to overcome. It appears that they have been overcome. In order for the value of this project to be spread, it would be useful if the authors could discuss the most difficult problems – the things that keep you awake at night – and how they were (successfully or not) addressed. Without that, if others try to implement what you have done, they will re-invent the wheel, with all the unanticipated problems.• Comparisons to others and overall success: While comparing to other systems would be dangerous, it would be useful to see a comparison to what existed before. The authors have strong take-home messages, and, while I have a gut-feel agreement with them, there is very little in the text to support these take-home messages. A comparison with what had existed before, and what the results were, would help to understand the overall quality of the program, and would lend support to the take-home messages.So, while the paper does have value, I would have like the authors to use it as an opportunity to share their detailed experience so that other could evaluate and build upon the lessons learnt.

Reviewer Expertise:

NA

No decision status is available

# Reviewer report: Reviewer response for version 1

**DOI:** 10.21956/mep.19737.r29578

**Published:** 2018-12-20

**Authors:** Trevor Gibbs

**Affiliations:** 1 AMEE

This review has been migrated. The reviewer awarded 4 stars out of 5 We frequently congratulate our outstanding students and universities are more often than not judged o the number of students they graduate- we support the good student. One feature that many Universities are poor at is supporting the struggling student. There is an acceptance that those struggling are struggling because they lack the academic standing, they are not up to scratch academically. All to often the students fails without any investigation of why they failed, despite them being "good enough" to enter into the University.I thought that this paper tackled an important area of the struggling student- their background and financial and social standing. I would have liked the authors to expand more on their three-pronged programme, simply because I am fascinated to find out more of how they achieved their outcomes. However, as the paper stands it begins to show a good insight into why students struggle and some ideas of how their learning process can be improved. I would recommend this to all of those involved in student welfare and curriculum planning.

Reviewer Expertise:

NA

No decision status is available
